# Utility and usability of a wearable system and progressive-challenge cued exercise program for encouraging use of the more involved arm at-home after stroke—a feasibility study with case reports

**DOI:** 10.1186/s12984-024-01359-0

**Published:** 2024-04-30

**Authors:** Jake Horder, Leigh A. Mrotek, Maura Casadio, Kimberly D. Bassindale, John McGuire, Robert A. Scheidt

**Affiliations:** 1 Biomedical Engineering, Marquette University and Medical College of Wisconsin, Milwaukee, WI USA; 2 Physical Medicine and Rehabilitation, Medical College of Wisconsin, Milwaukee, WI USA; 3 Biomedical Engineering, University of Genoa, Genoa, Italy; 4 Biomedical Engineering, Marquette University and the Medical College of Wisconsin, Engineering Hall, Rm 342, P.O. Box 1881, Milwaukee, WI 53201-1881 USA

**Keywords:** Stroke rehabilitation, Wearable devices, Patient-centered movement therapy, User experience, Motivation, Satisfaction

## Abstract

**Background:**

Understanding the role of adherence to home exercise programs for survivors of stroke is critical to ensure patients perform prescribed exercises and maximize effectiveness of recovery.

**Methods:**

Survivors of hemiparetic stroke with impaired motor function were recruited into a 7-day study designed to test the utility and usability of a low-cost wearable system and progressive-challenge cued exercise program for encouraging graded-challenge exercise at-home. The wearable system comprised two wrist-worn MetaMotionR+ activity monitors and a custom smartphone app. The progressive-challenge cued exercise program included high-intensity activities (one repetition every 30 s) dosed at 1.5 h per day, embedded within 8 h of passive activity monitoring per day. Utility was assessed using measures of system uptime and cue response rate. Usability and user experience were assessed using well-validated quantitative surveys of system usability and user experience. Self-efficacy was assessed at the end of each day on a visual analog scale that ranged from 0 to 100.

**Results:**

The system and exercise program had objective utility: system uptime was 92 ± 6.9% of intended hours and the rate of successful cue delivery was 99 ± 2.7%. The system and program also were effective in motivating cued exercise: activity was detected within 5-s of the cue 98 ± 3.1% of the time. As shown via two case studies, accelerometry data can accurately reflect graded-challenge exercise instructions and reveal differentiable activity levels across exercise stages. User experience surveys indicated positive overall usability in the home settings, strong levels of personal motivation to use the system, and high degrees of satisfaction with the devices and provided training. Self-efficacy assessments indicated a strong perception of proficiency across participants (95 ± 5.0).

**Conclusions:**

This study demonstrates that a low-cost wearable system providing frequent haptic cues to encourage graded-challenge exercise after stroke can have utility and can provide an overall positive user experience in home settings. The study also demonstrates how combining a graded exercise program with all-day activity monitoring can provide insight into the potential for wearable systems to assess adherence to—and effectiveness of—home-based exercise programs on an individualized basis.

**Supplementary Information:**

The online version contains supplementary material available at 10.1186/s12984-024-01359-0.

## Background

Stroke is a leading cause of disability and a growing public health concern. Approximately 9.4 million Americans had a stroke between 2017 and 2020 (an overall prevalence of 3.3%, which is expected to increase to nearly 4% by 2030) [1]. An increasing population of individuals surviving stroke poses a significant burden on social, economic, and health care systems because motor impairments that limit movement on one side of the body affect up to 80% of survivors [2]. Motor impairments significantly degrade quality of life by hindering activities of daily living and limiting community participation. Deficits in motor function often result from physical impairment, but can also arise from the behavioral phenomenon of learned nonuse whereby limb use is suppressed despite sufficient motor capacity [3]. Prolonged nonuse can lead to weakness and contractures that further exacerbate impairment. Therefore, a primary goal of rehabilitation after stroke focuses on promoting recovery of impaired movements. Exercise training is an effective tool in restoring motor function, even beyond the acute stage of recovery wherein most of the practical gains are typically seen [4, 5].

Although physical and occupational therapists commonly prescribe home exercise programs to manage residual sensorimotor deficits after stroke, adherence rates can be low thereby limiting the patient’s potential recovery [6, 7]. Home-based tele-rehabilitation programs with therapists providing intermittent supervision of the patient may help mitigate adherence issues [7] and demonstrate potential functional benefits [8, 9] that equal or exceed those provided by conventional face-to-face therapy (for reviews see [10, 11]). Augmenting home-based tele-rehabilitation programs with wearable technologies that monitor movement and provide feedback to patients and therapists has potential to improve stroke outcomes through increased intensity of therapy and adherence to rehabilitation programs [12, 13]; see also [9]. Despite its promise, wearable activity monitoring technology is not widely used by therapists in day-to-day stroke care in the clinic [14] or by patients at home. Barriers to adoption include lack of skills and knowledge of patients, not knowing what brand and type of monitor to choose (cf. [15]), and the skills, beliefs, and attitudes of individual therapists, which determine the current use of wearable technology [14]. Other factors, including user motivation and trust in the technology likely contribute to patterns of low adherence (cf. [16, 17]).

In recognition of this opportunity, there has been a recent explosion in the number and types of technologies proposed to promote adherence to home-based exercise programs for physical rehabilitation after stroke [18–29], for reviews see [30–32]. Most of these systems use micro-electro-mechanical sensor (MEMS) technology such as accelerometers and gyroscopes to monitor body movements in real-time and to derive summary statistics such as “activity counts” [22, 33, 34] or measures of movement smoothness [23]. Other approaches to motion tracking are possible such as visual image processing systems cf. [35] but lack the portability and convenience of wearable systems.

The most common application of wearable technologies is the monitoring and assessment of the quantity and quality of movement [18, 26, 36–40]. Wearable technologies can also provide helpful cues (i.e., reminders, nudges) to perform activities such as exercises prescribed as therapy [19, 20, 24, 25, 27, 28, 41]. In one exemplar study, Holden and colleagues describe a wrist-worn system [24] that provided a vibratory stimulus to the more-involved arm when that arm’s activity level fell below a personalized threshold for a selected time window. If prompted, the participant was instructed to increase more-involved arm movements, ideally by performing pre-selected activities from a self-directed repetitive functional task practice program [19]. The study protocol also included twice weekly meetings with a study therapist to download acceleration data and to provide performance feedback to the participant. Across seven participants and the 4-week program, this system provided only a small number of cues per day (median = 4), although mean arm activity increased following prompts by 11% to 29%. The authors conclude that personalized prompts delivered by a wrist-worn accelerometer may enhance self-directed arm activity after stroke [19]. However, for technologies to be adopted by clinicians and their patients, the systems must not only have practical utility, but they must also provide a positive user experience within the context of their intended use scenarios cf. [20, 27, 29].

To date, wearable rehabilitation exercise systems have prompted users to increase activity relatively infrequently, such as once every 10 min [25], once an hour [24] or less frequently [24, 27]. User feedback indicates that they desire flexible exercise schedules and well-defined exercise recommendations with graded challenge levels based on ability [27]. In the current study, we sought to assess the utility, usability, and user experience of a wearable exercise cueing and monitoring system designed to promote high-dose (60 cues per 30-min exercise session, 3× per day), graded-challenge exercise at-home with a small cohort of hemiparetic stroke survivors. Our clinician-designed exercise program was motivated by the fact that many survivors of stroke are ineligible for interventions such as constraint-induced movement therapy (CIMT) [42, 43], which is suitable only for participants retaining substantial motor capacity (e.g., residual wrist extension). Our novel exercise program seeks to engage patients in high-dose activities while accepting of a broad range of impairment levels, ranging from an inability to move the more-involved arm and hand to the ability to move that limb independently. Our study included a wide age range of participants (29 to 63 years old), a wide range of time post-stroke (2 to 21 years), and varying living situations (i.e., living alone, living with family, or in assisted care settings) to demonstrate how such a system can be used by different populations. Quantitative movement data was derived from motion trackers worn on both wrists. End-user feedback was collected through well-validated quantitative surveys that assess key aspects of the subjective user experience. Additional qualitative feedback was collected through questionnaires and informal discussions designed to solicit recommendations for future system modifications or improvements. After summarizing our findings, we provide two individual case reports that demonstrate how patients with different levels of residual motor capacity chose to engage with the cued exercise program in their home setting.

## Methods

Eight survivors of hemiparetic stroke were recruited from the Froedtert Hospital and Medical College of Wisconsin communities. Inclusion criteria included: age greater than 18 years; history of a single hemiparetic stroke; ability to follow two-stage instructions. Exclusion criteria included: inability to give informed consent; loss of upper-extremity function in both arms; and inability to don the wearable devices due to a lack of independent physical ability with also lack of personal support care to assist with donning the devices each day. All procedures received institutional review and approval from Marquette University and the Medical College of Wisconsin. All recruited participants provided written, informed consent to participate in this study in accord with the Declaration of Helsinki (2013 revision). The protocol was explained to each participant along with its potential risks, and each was informed of their right to discontinue participation in the study at any time.

All participants showed impaired motor and/or sensory function contralesional to their stroke injury (Table 1). Two of the participants did not complete the entire 7-day study protocol: participant 3 attempted to begin the protocol but was unable to balance the time commitment needed for the study against that needed for work; participant 6 completed 2 days of the protocol but then lost the equipment. The remaining participants completed the entire protocol successfully. We excluded from the analyses described below all data from the two participants who did not complete the entire study.

**Table 1:** Stroke survivor demographic data

Subject	Age	Sex	MoCA	Years post-stroke	Stroke type	Stroke location
1	29	M	26	21	H	L BG
2	36	F	25	3	H	L BG
3^a^	28	F	27	1	I	R MCA
4	50	F	25	5	I	L MCA
5	63	F	26	2	I	L MCA
6^a^	60	F	29	24	I	L MCA
7	63	F	30	8	I	R MCA
8	51	F	26	5	H	R MCA

*H* hemorrhagic, *I* ischemic, *L* left, *R* right, *BG* basal ganglia, *MCA* middle cerebral artery

^a^Did not complete study

### Materials

**Fig. 1:**
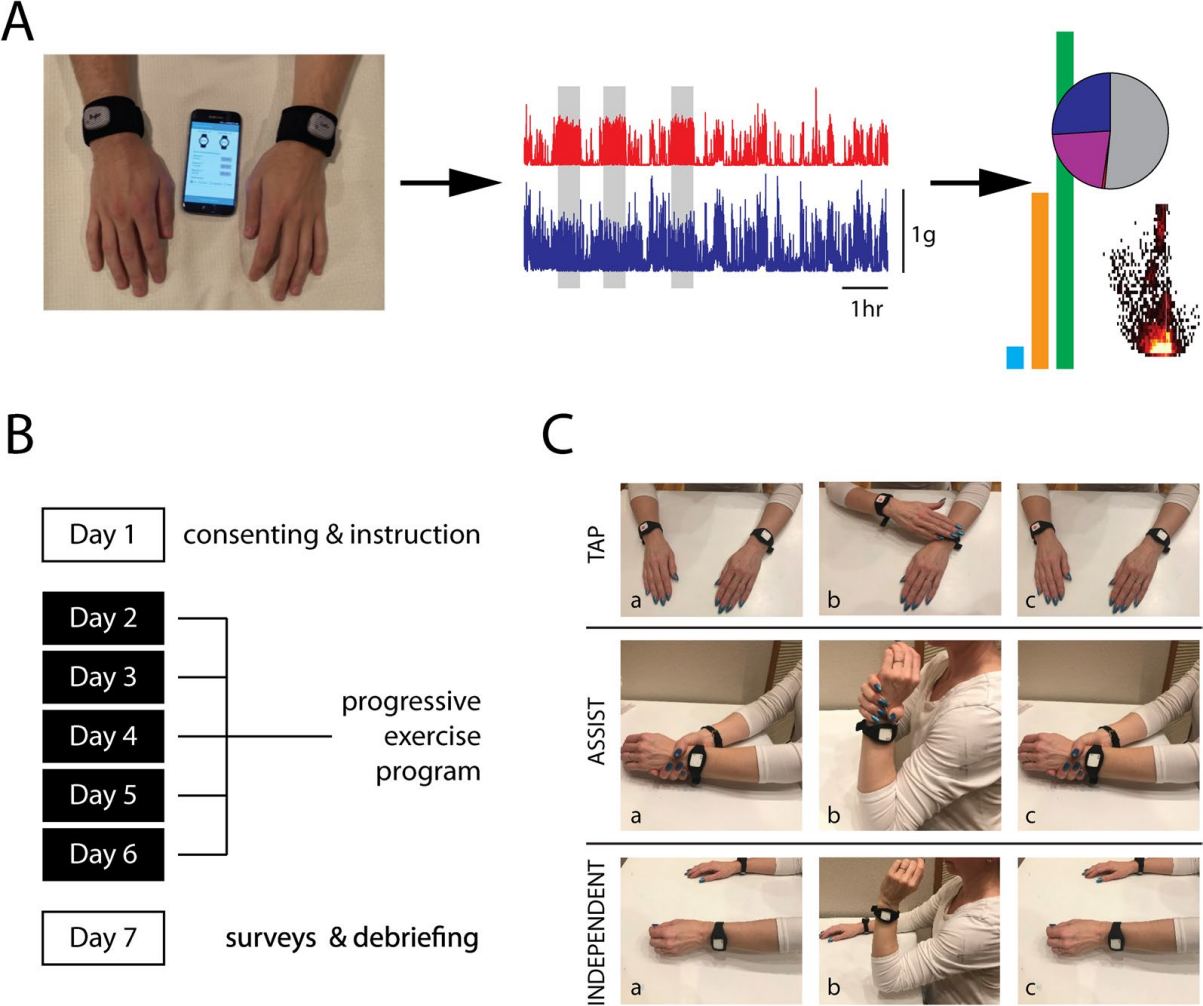
Study design. **A** Information workflow from wearable devices and smartphone app to data post-processing for sensor data synchronization and interpretation. Left: the devices record motion data and communicate via a Bluetooth connection with a custom application to generate exercise cues. Center: accelerometer data from both limbs is synchronized and processed to obtain motion profiles as a function of time. Right: motion data is further processed to obtain objective measures of bilateral arm use. **B** Study timeline. **C** Cued exercises ranging from easiest (Tapping; top) to more challenging (Independent; bottom). Tap: when cued, the participant was to use their LA arm and hand to tap the wearable device on their MA limb’s wrist. Assist: when cued, the participant was to grasp the wrist of their MA arm with their LA hand and to use the LA limb to flex and then extend their MA elbow. Independent: when cued, the participant was to perform independent flexion–extension movements of their MA arm

The activity monitoring systems used in this study comprised: two low-cost MetaMotionR+ wearable activity monitors (MBIENTLAB; San Francisco, CA) with one worn on the more-affected (MA) contralesional arm and one on the less-affected (LA) ipsilesional arm; a low-cost Android smartphone with Bluetooth 5.0 capability (Google Pixel 5a); and a custom smartphone app that managed exercise scheduling and data management (Fig. 1A). Charging cables, USB power adapters, and Velcro watch bands were also included as supporting accessories for the core system components.

The MetaMotionR+ devices each include a 3-axis accelerometer, gyroscope, magnetometer, and an ARM® Cortex™ M4F CPU that can provide 9-axis sensor fusion yielding an orientation vector that can be recorded at sample rates up to 400 per second. For this study, we only utilized the 3-axis accelerometers (16-bit resolution: 1.2 × 10^−4^ g) to minimize energy consumption. We limited the sampling of motion from each wrist-worn MetaMotionR+ device to 25 samples per second to allow each device to store more than 8 h of time-stamped data. The devices also include a vibrating coin motor similar to those that provide tactile feedback in cell phones; we used the motors to provide vibrotactile cues to the participants when it was time to perform their exercises.

The MetaMotionR+ devices also have Bluetooth Low Energy capability, which allows them to communicate robustly with a dedicated smartphone app. The application performs a variety of functions that include: facilitating the interactive selection and scheduling of specific exercises to be performed each day; setting the strength of vibrotactile exercise cues per user preferences; automatically transferring deidentified data from the two wrist-worn MetaMotionR+ devices to a secure cloud server at the end of each day; automatically managing the Bluetooth connection and handling connection interruptions; and monitoring the battery charge for each wearable device.

### Protocol design

A 7-day experimental protocol was designed to test the utility and usability of a low-cost wearable system that challenges participants with a progressive set of in-home idle-time exercises that can be customized to their individual upper-limb physical ability and schedule (Fig. 1B). Study activities included administrative setup, exercise and activity monitoring, and administrative wrap-up:

#### Day 1: consenting, set-up, and instruction

After an initial phone screening, two members of the study team met with the participant and a caregiver at their home or at an agreed-upon public location to conduct informed consent. Then, the study members provided written and verbal instructions on how to set up and use the wearable exercise cueing system. Specific instructions were provided regarding how to don and doff the two wrist-worn activity monitors (one per arm), how to use the dedicated smartphone app, and how to charge the three devices at the end of each day. The participant practiced putting on the wearable devices, connecting the devices to the phone, and setting specific exercise session start times. A mock exercise session was also started for the participant to experience the vibrotactile cues from the wearable devices and to practice making the exercise movements. This full demonstration allowed the participant to become familiar with the system and to ask any questions while the research team was present. The researchers also reviewed the documentation provided to the participant, including the app’s user guide, the graphical exercise instructions, and a daily feedback form to be completed at the end of each day of cued exercises. At the end of the introduction, the researchers and the participant agreed upon a time the following morning for a telephone check-in to assist with setting up the first day of exercise monitoring. To encourage compliance with the study protocol and to allow the research team to assist the participant with scheduling the daily exercises, check-in calls were also scheduled for the morning and night of each day of the progressive exercise program. Another function of these calls was to provide support addressing any technical issues with the system as they might arise. If participants felt comfortable navigating the system without the daily check-in calls, they were instead given the option to reach out to the research team only as necessary.

#### Days 2–6: progressive exercise program and passive activity monitoring

This study stage gave each participant experience interacting with the wearable exercise cueing system. It also allowed assessment of the in situ utility of the wearable system. Each day typically started with the check-in call, which allowed the study team to assist the participant and/or caregiver in selecting which of three upper extremity exercises (described below) to perform that day, in scheduling three 30-min bouts of exercise to be performed that day, and in setting the desired intensity of vibrotactile exercise cues, which were applied by the wrist-worn devices. After setting up the system, each participant was encouraged to go about normal routines while wearing the activity monitors so that the system could capture typical arm usage throughout activities of daily living.

Three minutes before the beginning of each exercise period, the wearable devices gave the participant a warning notification consisting of three short vibrations to indicate an exercise period would start soon. During each 30-min exercise period, both wrist-worn devices provided short (1 s) vibrotactile stimuli spaced 30 s apart. Thus, the participant was to perform 60 cued exercises within each of three, 30-min exercise blocks, for a total of 180 movements over a total period of 90 min. At the end of each exercise period, the system reverted to its “silent monitoring” mode by continually collecting acceleration data from the participant without initiating any haptic cue or notification. The participant was encouraged to distribute the exercise windows throughout an 8-h period of upper extremity activity monitoring, which could also be scheduled. The system was programed such that exercise periods had to be separated in time by at least 30 min. Exercise periods also could not be scheduled within the first and last 30 min of the 8-h session. The participant was asked to keep the study smartphone in close proximity through each day of activity monitoring.

At the end of each day, the smartphone automatically downloaded the anonymized and time-stamped acceleration data from the two wrist-worn activity monitors, and then uploaded the data to a secure cloud server for remote access by the study team. Each participant was also asked to fill out an end-of-day self-evaluation form that used a visual analog scale (range: 0 to 100) to indicate how well the exercises for that day were completed. There was additional space on the form for the participant to document any questions or comments. At the agreed upon time, the researcher called the participant for the second daily check-in to address any issues that may have come up during the day, to discuss the participant’s exercise progress, to remind participants to recharge all three study devices, and to schedule check-in calls for the following day. Five participants felt sufficiently confident with all aspects of the system after just 1 or 2 days of check-ins that they indicated their desire to forgo daily check-ins and to reach out to the study team only if they had issues or concerns.

#### Day 7: debriefing and system retrieval

After the participant had used the progressive exercise cueing system for 5 days, the study team re-visited the participant to retrieve the study materials and to conduct three surveys of system usability and subjective user experience (see Table 2):

- The System Usability Scale (SUS) [44] is a quick, reliable tool for measuring the usability of a system, i.e., its “appropriateness to a purpose within a given context” [45]. We assessed the usability of our exercise cueing and monitoring system in the context of encouraging therapeutic arm activity at home after stroke. The SUS is easy to administer, valid, can be used on small sample sizes with reliable results, and can effectively differentiate between usable and unusable systems. Scores range from 0 to 100; scores greater than 68 indicate passable usability [45].
- The Intrinsic Motivation Inventory (IMI) [46] is a questionnaire commonly used to assess subjective experience of motivation during activities [47]—including stroke rehabilitation, where it has been used to assess perceived motivation during interventions [48, 49]. We used a 37-item version of the questionnaire that included questions spanning six dimensions: interest/enjoyment, importance/effort, value/usefulness, perceived choice, perceived competence, and felt pressure/tension. The interest/enjoyment subscale is generally considered to be the self-report measure of intrinsic motivation. An average subscale score equal to 4 or greater would indicate that participants found the system motivating to use.
- The Quebec User Evaluation of Satisfaction with assistive Technology (QUEST) [50] was designed to evaluate user satisfaction with a wide range of assistive technologies [51]. QUEST provides a means to document the real-life benefits of assistive technologies and to justify the need for such devices. The 12-item version we used assesses user satisfaction in terms of the system’s physical characteristics (e.g., size, weight), physical and cognitive fit (e.g., physical comfort, ease in adjusting, ease of learning), functional characteristics (e.g., how successfully the device performed), and social support (e.g., reactions of others during use). It also includes a list of 12 items from which the user was to select three as the most important for providing a satisfactory user experience.

**Table 2:** Summary of user experience surveys provided to each subject

	SUS	IMI	QUEST
Items	10	37	12
Scoring	1–5 scale: strongly disagree to strongly agree	1–7 scale: not at all to very true	1–5 scale: not satisfied at all to very satisfied
Subcategories	None	• Interest/enjoyment • Effort/importance • Value/usefulness • Perceived choice • Perceived competence • Pressure/tension	• Smartphone app and wearable devices • Training and assistance
Sample questions	• I thought the system was easy to use • I think that I would need the support of a technical person to be able to use this system • I found the various functions in the system were well integrated	• It was important to me to do well at the exercises • I put a lot of effort into the cued exercises • I think I did pretty well with the exercises compared to others who may also be using the same system	• How satisfied are you with how easy it is to use the smartphone app? • How satisfied are you with how comfortable the wearable devices are? • How satisfied are you with the troubleshooting of issues provided by the researchers during setup and use of the app and devices?

*SUS* System Usability Scale [44], *IMI* Intrinsic Motivation Inventory [46], *QUEST* Quebec User Evaluation of Satisfaction with assistive Technology [50]

Each participant was provided a small monetary incentive upon returning the activity monitors and smartphone.

### Progressive exercise stages

On study days 2 through 6, each participant performed one of three simple upper extremity exercises designed to progressively challenge use of the more-affected (MA) arm.

#### Stage 1: tapping

This exercise stage served to familiarize the participant with making goal-oriented movements in response to vibrotactile cues and to focus attention onto the MA arm. In response to each vibrotactile cue, the participant used the less-affected (LA) hand to tap the wearable device on the involved limb’s wrist (Fig. 1C; top row: TAP). Following the single tap, the participant returned the LA arm to a neutral position. This exercise requires no functional movement of the MA arm, but instead serves to focus the subject’s attention to that limb. As such, this exercise should be able to be performed by individuals independent of the severity of impairment of the MA upper extremity [52].

#### Stage 2: assisted movements

The participant used the LA arm to assist flexion–extension movements of the MA elbow (Fig. 1C; middle row: ASSIST). In response to each cue, the participant grasped the wrist of the MA arm with the LA hand and use it to assist the MA elbow through a flexion–extension cycle. The participant was advised to go through as much of the affected limb’s range of movement as possible.

#### Stage 3: independent movements

In the final exercise stage, the participant performed independent flexion–extension movements of the MA elbow (Fig. 1C; bottom row: INDEPENDENT). These movements were performed without the assistance of the LA limb. In response to each cue, the participant was to execute—if possible—a single flexion–extension movement of the MA elbow without assistance using as much of its range of motion as possible.

We initially intended to progress each participant through the first two stages on Day 2 (TAP) and Day 3 (ASSIST), devoting Days 4, 5, and 6 to the more difficult (INDEPENDENT) stage 3 exercise. Four of the six participants who completed the 5-day protocol were able to follow this order (Table 3). The remaining participants expressed the desire to personalize their progression by reverting to an easier exercise after experiencing a more difficult exercise.

**Table 3:** Progression of exercise stages for all participants

Subject	Day 1	Day 2	Day 3	Day 4	Day 5
1	Tap	Assist	Independent	Independent	Independent
2	Tap	Assist	Independent	Independent	Independent
3^a^	–	–	–	–	–
4	Tap	Assist	Tap	Independent	Assist
5	Tap	Assist	Independent	Independent	Independent
6^a^	Tap	–	–	–	–
7	Tap	Assist	Independent	Independent	Independent
8	Tap	Assist	Assist	Assist	Assist

^a^Did not complete study

It is worth noting that the simple exercises designed for this feasibility study were intended only as an example of exercises able to provide progressive-challenge to MA arm movement. These exercises facilitated demonstration of accelerometry’s ability to discriminate different UE activities, as will be shown below in two case studies. Future implementations of the system could of course cue other exercises specific to the participant’s abilities and home exercise program as designed by their therapy team.

### Data analysis

**Fig. 2:**
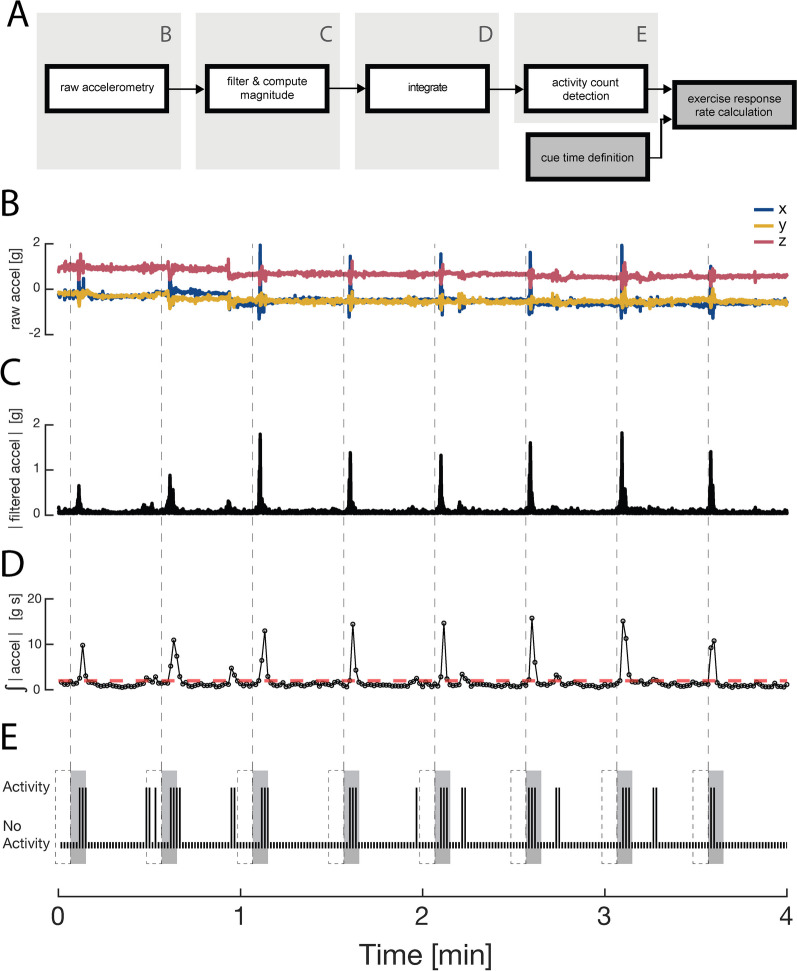
Accelerometry data processing stages. **A** Data processing flow diagram for the cue response detection algorithm. The timestamp of a given vibrotactile cue was used as input to determine if arm activity was detected within a 5 s time window following each cue. **B** Raw accelerometry data for each dimension (X, Y, Z) recorded over a selected 4-min window from one arm of a selected subject. Dashed vertical lines indicate the timing of vibrotactile cues. **C** Filtered and rectified accelerometer data for each dimension (overlapped). **D** Acceleration magnitude summed within each 1-s epoch. Red dashed horizontal line: the specific activity threshold used in this study. **E** Limb activity element within each 1-s epoch for this representative 4-min window. Also highlighted are the 5-s windows immediately preceding (dashed boxes) and following (gray boxes) each cue (vertical dashed lines)

We computed three primary outcome variables pertaining to system utility. We defined *system uptime* as the overall amount of time that the two MetaMotionR+ devices recorded accelerometry data during each day’s scheduled monitoring period. We computed the *vibrotactile cue rate* by dividing the number of cue indicators stored in each file (cf. Fig. 2B, vertical dashed lines) by the expected value of 180 cues per day. Several post-processing steps were needed to compute the third variable, *exercise response rate*. Figure 2 outlines (Fig. 2A) and demonstrates these steps (Fig. 2B–E).

Each of the MetaMotionR+ devices output unfiltered accelerometry values to respective data files for each arm. New files for both arms were generated each day, with each file containing that day’s acceleration values for the X, Y, and Z axes in gravitational units (1 g = 9.8 m/s^2^; Fig. 2B). The files also included time-stamped indicators of when vibrotactile cues were provided by the MetaMotionR+ devices. The accelerometry values along each axis were filtered using a bandpass window of 0.25 Hz to 2.8 Hz. This frequency window was similar to those used in previous accelerometry-based studies for upper extremity motion and has been shown to filter out the static effect of gravity as well as extraneous motion and noise [53, 54]. We then computed the magnitude of the limb acceleration using the Euclidean norm (Fig. 2C). The resulting acceleration magnitude data were then summed within each 1-s epoch to yield a time series of each arm’s acceleration magnitude for every second of the 8-h monitoring period per day (Fig. 2D). We then transformed the acceleration magnitude values into an intermediate variable called *activity counts* using a thresholding approach similar to those described by Bailey et al. [22] and Uswatte et al. [22, 33]. Specifically, each 1-s epoch with a vector magnitude greater than or equal to 2 g (Fig. 2D, red horizontal line) was considered a 1-s epoch with activity (Fig. 2E); epochs with a magnitude less than 2 g were considered to have no activity. The sum of active epochs over a given time interval represented the total duration of upper-limb activity for that specific interval. We then computed *exercise response rate* by counting the number of vibrotactile cues immediately followed (within 5 s; Fig. 2E, grey boxes) by a period of arm activity greater than or equal to 1 s, and then dividing by the actual number of cues provided. We similarly computed a *baseline activity rate* by counting the number of vibrotactile cues immediately preceded (within 5 s) by a period of arm activity greater than or equal to 1 s, and then dividing by the actual number of cues provided. We compared the two rates to assess the ability of vibrotactile cues to instigate upper extremity exercise. For Stage 1 (Tapping), we only considered LA activity because the exercise typically elicited arm accelerations biased strongly toward the LA arm. For Stage 2 (Assisted Movements), we required activity within both arms during the response interval because the exercise required synchronous motions of the two arms. For Stage 3 (Independent Movements), we only considered activity within the MA arm. We did not eliminate activity counts here when there was concurrent activity on the LA side because participants were not instructed to forego unrelated movement or activity during exercise periods; consequently, some level of bimanual background activity was expected.

We also computed a number of secondary performance variables based on the kinematic data; we report these measures for two selected participants as case studies. Subjects 1 and 8 were selected as specific cases because they exhibited the least and the most MA-arm impairment, respectively, across the whole cohort at the time of this study. Activity counts and acceleration magnitudes from each arm were combined to obtain three measures of bilateral arm use; these included bilateral arm use ratio, bilateral acceleration magnitude, and bilateral magnitude ratio. Specifically, we calculated the *bilateral arm use ratio* by dividing the total activity count of the MA arm by the total activity count of the LA arm within specific time windows. The bilateral arm use ratio assesses arm usage within a given time window, with a value of 1 indicating equal usage between affected and unaffected arms and a value of 0 indicating no affected-side usage. We computed *bilateral acceleration magnitude* by summing the acceleration magnitudes across the two limbs within each 1-s epoch. The bilateral acceleration magnitude is an indication of movement intensity across both limbs. We then computed a measure we called *bilateral magnitude ratio* by taking the natural log of the value obtained by dividing the MA arm’s acceleration magnitude by the LA arm’s magnitude within each 1-s epoch. The bilateral magnitude ratio differs from the bilateral arm use ratio in that the resultant value of the magnitude ratio provides a measure of relative movement intensity that is absent from the arm use ratio, which is predominantly concerned with arm use as a function of time.

Finally, we computed three primary outcome variables pertaining to subjective user experience using the SUS, IMI, and QUEST surveys. Survey scores were computed using each survey’s standard scoring method. For the SUS, negatively worded items were subtracted from the maximum scale value of 5 before summing all items. The sum of all ten item scores was multiplied by 2.5 to give an overall score within the range: 0 to 100 [44]. Our 37-item version of the IMI was constructed using language specific to determining motivation related to performing the exercises in response to haptic cues. Responses within each of the six subscales were averaged for each participant. To score the QUEST, we computed average response within each subscale (device, services) for each participant.

### Statistical hypothesis testing

We tested two hypotheses. We first hypothesized that the system would have utility in the sense that it will reliably evoke exercises on cue (Hyp 1). We then hypothesized that users of the system will have a positive subjective experience using the devices in a home setting (Hyp 2). To test Hyp 1, we used paired, one-sided t-test to compare the *cue response rate* within the 5-s window following each cue to the *background activity rate* measured in the 5-s windows immediately preceding each cue for each participant. To test Hyp 2, we used one-sample, one-sided t-tests to compare each survey’s mean results for the participant group to nominal values based on each survey’s scoring methodology. The SUS scores were compared to 68 as a threshold for acceptable usability; scores above this threshold are generally regarded as indicative of positive system usability [45]. Without standardized IMI and QUEST scores to compare to, the scores above the midpoints of the respective 7- and 5-point Likert scales were used to demonstrate a baseline level of acceptability for each survey [55]. Because small sample sizes challenge assumptions underlying t-test analyses, we used Wilcoxon signed rank test to confirm the results of the planned t-tests. Altogether, the nonparametric analyses yielded the same pattern of statistical significance as described for the planned t-tests below. Statistical analyses were performed with GraphPad Prism software version 9.0 (GraphPad Software, Inc., San Diego, CA). Results were considered significant at p < 0.05 for all tests.

## Results

### System performance

**Fig. 3:**
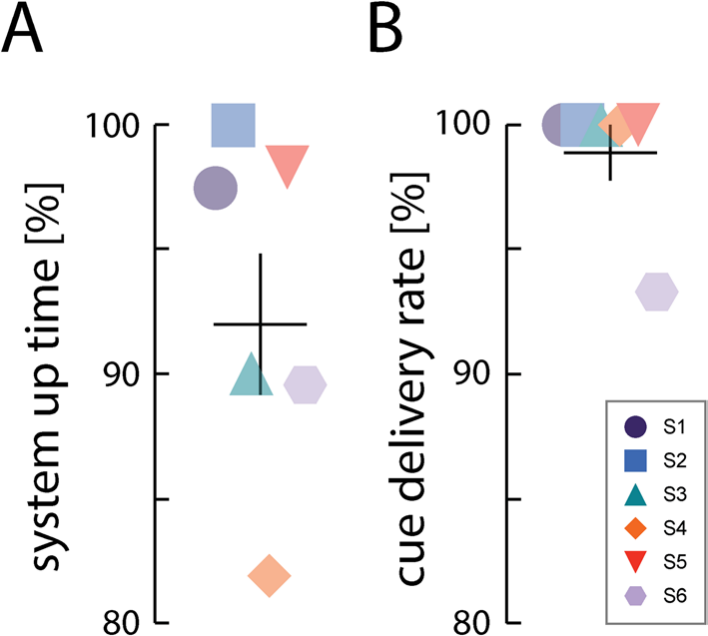
System performance over the duration of the study. **A** System uptime as defined as hours of activity data collected per subject, as a percentage of the 80 total hours collected across the two devices for each participant. **B** Cues delivered by the system during the scheduled exercise windows presented as a percentage of the 895 total cues given to each participant. Horizontal bars depict the cohort mean; vertical error bars depict ± 1 SEM

For the six participants who completed the study protocol, the smartphone maintained its Bluetooth connections with the wearable devices and recorded accelerometry data for 92% ± 6.9% (mean ± SD; here and elsewhere) of the 80 intended monitoring hours per participant (Fig. 3A). Five of the six participants reported full adherence to wearing the devices for 8-h per day; only one participant reported taking off the wrist devices for 1.5 h on 1 day. The systems experienced at least one Bluetooth disconnect event per participant, where the wearable device(s) lost connection with the phone for a brief period before automatically reconnecting. Most of these disconnect events were less than 1 min long. Other gaps in data collection resulted from battery charging issues; three participants failed to charge the wearable devices overnight, which resulted in at least one of the devices running out of charge during data collection. However, all accelerometry data were time stamped, which facilitated documentation of disconnection and battery issues.

The system reliably delivered exercise cues according to schedule. Five participants received the full number of exercise cues at the self-scheduled times. Only 1 participant did not receive self-scheduled exercise cues for one 30-min session due to a battery charging issue. The remaining two participants who experienced battery-related data loss nevertheless received all the scheduled cues because the device that retained charge was able to provide cues. The system’s rate of successful cue delivery was 99% ± 2.7% across participants (Fig. 3B).

**Fig. 4:**
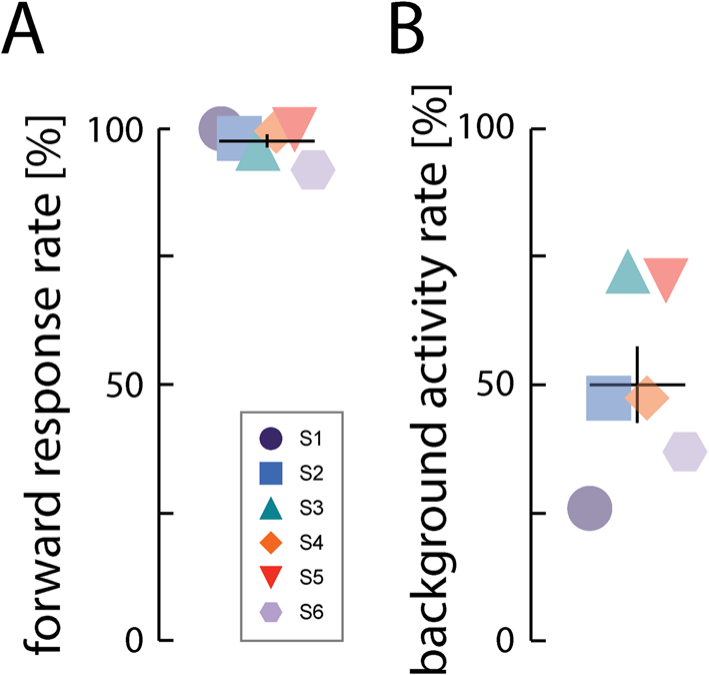
Mean activity rates. Activity rates calculated **A** in the 5-s cue response window immediately following each cue (i.e., the cue response rate), and **B** in the 5-s window immediately preceding each cue (i.e., the background activity rate). Horizontal bars: cohort mean; vertical error bars: ± 1 SEM

The system also was effective in eliciting responses to the exercise cues; across participants, an activity was detected within 5-s of the cue 98% ± 3.1% of the time (Fig. 4A; forward response rate). By contrast, far lower levels of activity were detected in the 5-s windows immediately preceding each cue; these background activity rates ranged from 26 to 72% across participants (Fig. 4B). Mean post-cue activity rates were significantly greater than pre-cue rates (paired, one-sided t-test: *t(5)* = *6.368*; *p* = *0.0007*). Background activity did not affect the capability to respond to cues because each participant increased their activity rate in response to the cues. These results confirm our hypothesis that the system has functional utility.

### User experience

**Fig. 5:**
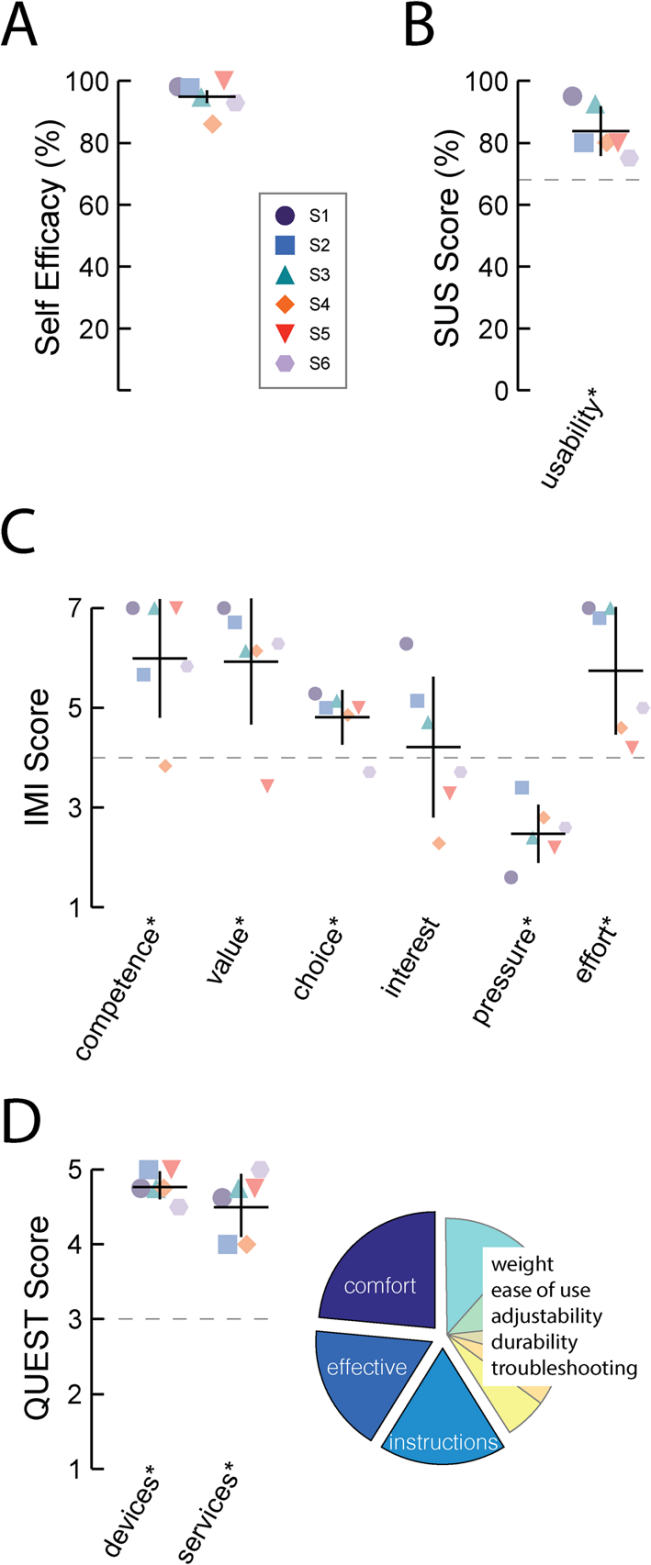
Post-study survey responses. **A** Self-efficacy as assessed via visual analog scale at the end of each day. **B** System Usability Scale, SUS. A score of 68 is considered to reflect passable usability. **C** Intrinsic Motivation Inventory, IMI. Sub-scores range from 1 to 7. Higher scores are associated with a more positive response to each category, except for the “pressure” subscale, where lower scores reflect a better user experience (e.g., feeling less pressured). **D** Quebec User Evaluation of Satisfaction with assistive Technology, QUEST. Left: sub-score scales ranges from 1 to 5, with higher scores indicating a more satisfactory user experience. In panels **B**–**D**, the horizontal dashed line indicates the threshold value indicating a positive user experience. Assessments with mean responses differing significantly from the threshold value are labeled with an asterisk (*one-sample t-test: p < *0.05)*. Right: aspects of the user experience identified as important for promoting a positive user experience; labels for factors that did not achieve top three ranking are listed in order of decreasing ranked importance (corresponding to the grouped pie chart sections in clockwise order). Horizontal bars: cohort mean; vertical error bars: ± 1 SEM

The system also elicited positive user experiences as evidenced by participant responses on surveys of subjective opinion. Across days and participants, end-of-day assessments of self-efficacy (ability to complete cued exercises) averaged 95 ± 5.0 on a visual analog scale that ranged from 0 to 100 (Fig. 5A). Participants also provided comments on the end-of-day feedback form that reflected positive experiences. One participant commented on the exercises: “I completed all three assignments. It was very easy… it’s a great exercise.” Another participant noted that the system “Worked great! Very confident (100%) data was collected correctly.” These comments were consistent with the survey on system usability (Fig. 5B). SUS survey scores averaged 84 ± 8.0 out of 100 across participants. We used planned, one sample t-test to compare the group mean to a threshold score of 68 (considered acceptable usability) and found that the system did indeed demonstrate acceptable usability in the home setting *(t(5)* = *4.808*; *p* = *0.00*24).

Responses on the Intrinsic Motivation Inventory (IMI) reflected strong levels of *perceived competence*, *value*, *importance*, and *perceived choice* when using the system (Table 2). One-way, one-sample t-tests confirmed that average responses in each of these categories significantly exceeded the threshold value of 4 corresponding to a positive user experience (t(5) ≥ 3.316; *p* ≤ *0.0106* in each case; Fig. 5C). The category of *pressure* had an average response that was significantly lower than 4, which also reflected a positive user experience (t(5) = 6.090; *p* = *0.0008*). By contrast, some participants found the system to be more motivating to use than others; nevertheless, the average *interest* score (4.2 ± 1.4) tended to exceed the threshold value of 4, suggesting that the system could be considered nominally motivating in the home setting.

Responses on the Quebec User Evaluation of Satisfaction with assistive Technology (QUEST) reflected a high degree of user satisfaction with the devices themselves and in the training and troubleshooting support received throughout the study (Fig. 5D). Average responses on the *devices* subscale (4.8 ± 0.19) and the *services* subscale (4.5 ± 0.42) both exceeded a threshold value of 3, corresponding to positive user satisfaction (one-way, one-sample t-test; *devices*: t(5) = 23.32; *p* < *0.0001*; *services*: t(5) = 8.840; *p* = *0.0002*). Of the 12 items from which users were to select those being the most important for determining satisfaction, *comfort*, *system effectiveness*, and *instructions* were selected most often (Fig. 5D). The items *weight* and *ease of use* were each picked by 2 participants whereas *adjustments*, *durability*, *troubleshooting*, and *follow-up communication* were picked by 1 participant each. Device *dimensions*, *safety*, and *equipment delivery* were not selected by any of the participants. (Individual participant survey responses are tabularized and presented as Additional file 1: Table S1). Taken together, the user experience survey results confirm our hypothesis that the system would elicit positive subjective experiences when using the devices in a home setting.

### Case studies

The primary purpose of this study was to assess the feasibility of a wearable idle-time exercise cueing system in terms of its objective utility and subjective user experience in unstructured home settings. However, the accelerometry data suggest potential benefits that might accrue from increased doses of upper extremity exercise in the chronic stage of stroke recovery. In the paragraphs below we present two case studies highlighting this potential for the most capable and least capable participants in our study.

**Fig. 6:**
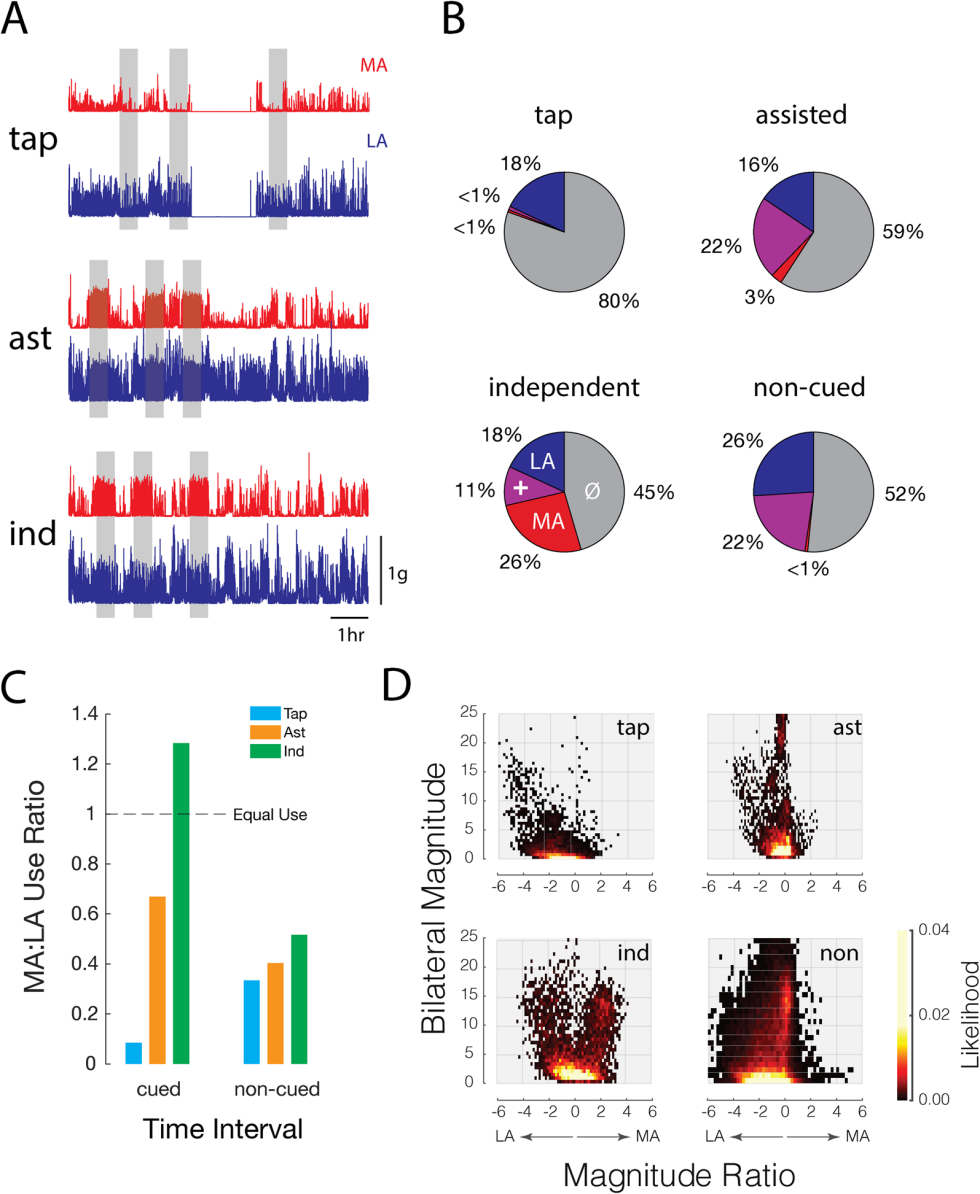
Case example Subject #1: a 29-year-old with partial independent motor function of the affected arm. **A** Filtered and rectified accelerometry vector magnitudes across all dimensions for 1 day of each exercise. Red: the more affected side (MA); Blue: the less affected side (LA). The gap in acceleration data between the second and third cued exercise windows on the first day (tap) corresponds to the participant doffing and donning the wrist devices before and after performing yardwork. **B** Arm usage for each exercise type within the cued exercise windows (tap, assisted, or independent) and within the silent monitoring periods (non-cued). +: both arms active at the same time; Ø: neither arm active. Each pie chart aggregates data within exercise periods of the same exercise type (tap, assisted, or independent) and across days for the silent monitoring periods (non-cued). **C** Arm usage ratios aggregated across days for each exercise type within the cued exercise windows and within the silent monitoring periods. A use ratio of 1 indicates equal usage from both sides. **D** Bilateral usage activity density plots aggregated across days for each exercise type within the cued exercise windows and within the silent monitoring periods

The first case (Subject #1) is a 29-year-old who, at the time of the study, was 21-years removed from a subcortical hemorrhagic stroke that had impacted the basal ganglia and had caused hemiparesis on the dominant side. This individual presented with a right-side motor deficit but was nevertheless able to move the affected arm independently. The participant followed the nominal 5-day progression of exercises, completing the tapping exercise on day 1, the assisted exercise on day 2, and the independent exercise on days 3 to 5. The accelerometry data shown in Fig. 6A were collected on days 1–3 and demonstrate the expected patterns of activity from both the LA arm (blue) and MA arm (red) throughout the day with each of the three different exercise modes. During each cued exercise window on Day 1 (tap), frequent accelerometry spikes of relatively high magnitude were observed in the LA arm, while the MA arm remained relatively stationary as the participant responded to each cue by tapping the MA arm with the LA hand. During the assisted exercise windows of Day 2 (ast), simultaneous accelerations of high magnitude were recorded from both devices as both arms moved together in response to each exercise cue. In the independent exercise windows of Day 3 (ind), the participant moved the MA arm in response to each cue, yielding cued MA arm acceleration profiles that were relatively consistent, and which appeared to exceed the non-cued MA accelerations (i.e., during silent monitoring periods) in both their magnitude (increased acceleration) and frequency (denser spikes of activity with less gaps in between movement). By contrast, the LA arm exhibited an amount of activity that neither increased nor subsided when the participant was performing the cued exercises with the MA arm.

The pie charts of Fig. 6B show the percentage of time each arm was active individually (Fig. 6B, red and blue shading), together (purple shading), and not at all (grey shading) during all of the cued exercise periods (tap, assisted, independent) and during silent monitoring periods aggregated across all 5 days (non-cued). During cued exercise periods, isolated usage of just the MA side was highest during the independent exercise stages (26%) and lowest in the tap exercise stage (< 1%) (Fig. 6B, red slice). By contrast, the percentage of time spent using just the LA side was consistent across exercise modes: 18% for tap, 16% for assisted, and 18% for independent (Fig. 6B, blue slice). The independent exercise stage elicited the highest amount of total activity with 55% of the window showing some measured level of activity from either the LA side, MA side, or both (red, blue and purple slices combined). As expected, these patterns of arm use were reflected in the ratios of overall MA arm use to the overall LA arm use—aggregated across days within each exercise stage—during the cued exercise windows; the ratio of MA to LA activity counts was highest during the independent exercise windows and lowest during the tap exercise windows (Fig. 6C, left). The use ratio was greater than 1.0 during the cued windows for the independent exercise days, indicating that the independent exercise was particularly effective in motivating use of the MA arm for this participant. The same trend of increasing arm use with increasing exercise challenge was also observed throughout the non-cued silent monitoring periods, with the highest use ratios recorded on days in which the independent exercise was performed and the lowest use ratio recorded on the day where the participant performed the tap exercise. These last results hint at a possible carry-over effect of the cued exercises on arm use throughout the rest of the participant’s day.

Finally, we visualized the intensity of bilateral arm activity using the density plots of Fig. 6D to verify that the cued exercises had the intended impact on the intensity of arm activity, not just on its frequency. Here we depict (as a heat-map) the likelihood that the total MA and LA arm acceleration magnitudes (i.e., bilateral magnitude) are biased toward the MA or LA sides (i.e., magnitude ratio) during any given 1-s interval. The four density plots are presented using data aggregated (across days) from each of the respective cued activity windows for each exercise and for the non-cued periods. During the cued exercise window on Day 1 (Fig. 6D, tap), arm use was biased toward the LA arm (i.e., negative magnitude ratios) with relatively few instances of high total arm acceleration (i.e., low likelihood of data points with high y-axis values of bilateral magnitude). By contrast, the density plot derived from acceleration data recorded during the cued exercise windows on Day 2 (Fig. 6D, ast) was more symmetrically distributed about the vertical line corresponding to a magnitude ratio of 0. This exercise also elicited a greater likelihood of bilateral arm activity exceeding 5 gravity-seconds along that midline, reflecting a “balance” in activity across the two arms. Wrist accelerations during the cued exercise windows on Days 3–5 (Fig. 6D, ind) were biased toward the MA arm, with a “hot spot” of activity centered on a magnitude ratio of 2 and a bilateral magnitude of 15 gravity-seconds, which was not present for any other cued exercise window or for the non-cued silent monitoring periods (Fig. 6D, non).

The second case (Subject #8) is a 51-year-old who, at the time of the study, was 5-years removed from a large-scale hemorrhagic stroke impacting the entire right MCA territory, causing significant left-side spastic hemiplegia. The participant could not move the MA arm independently but was able to do so with assistance from the LA arm. The participant completed the 5-day protocol performing the tap exercise on Day 1 and the assisted exercise on Days 2 to 5.

**Fig. 7:**
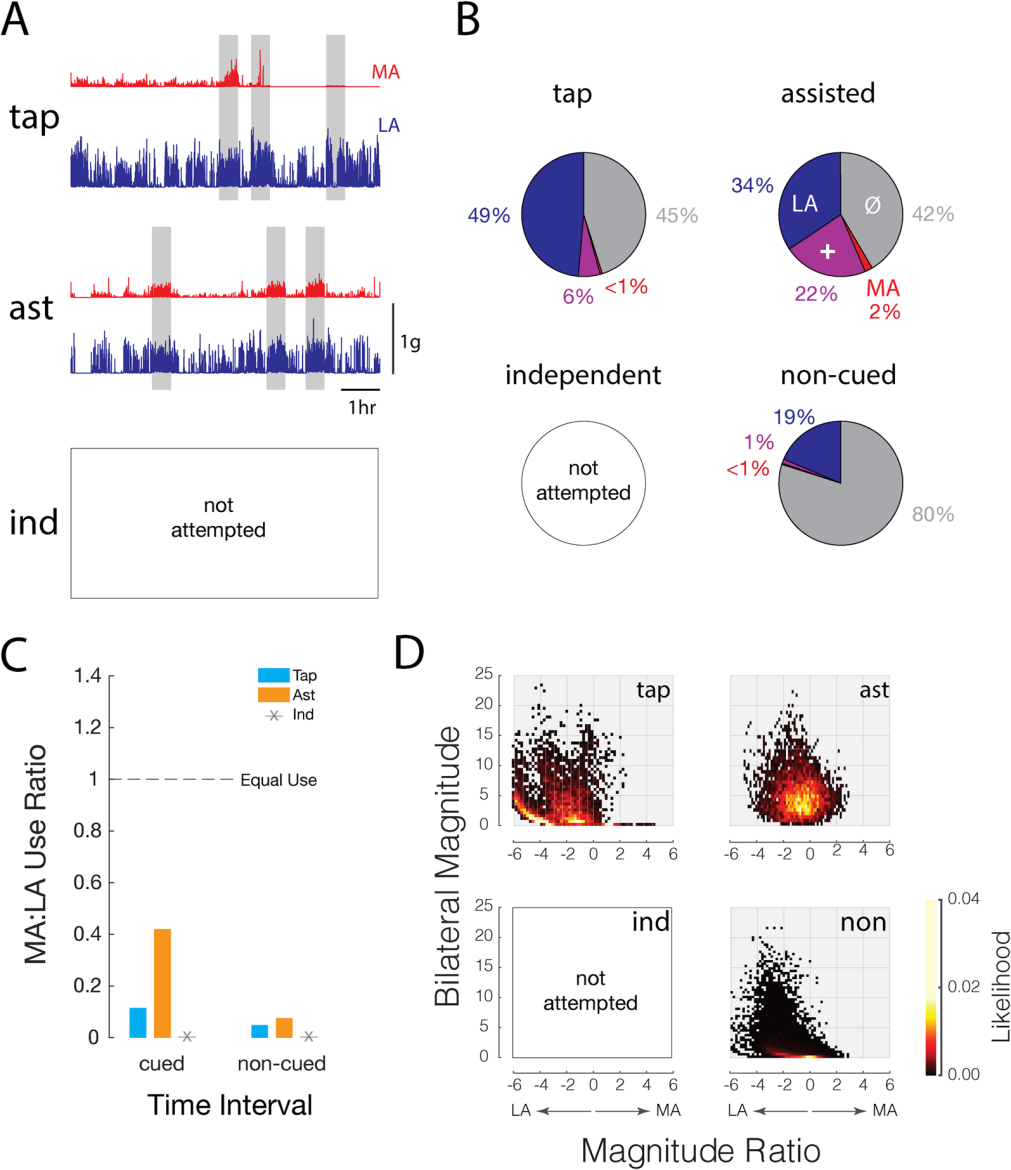
Case example Subject #8: a 51-year-old with effectively no independent function of the affected arm. Panel descriptions as for Fig. 6

This participant’s motor deficit is reflected in a notable imbalance in acceleration magnitudes recorded from the activity monitors worn on the MA and LA arms (Fig. 7A). Frequent spikes of high magnitude accelerations were observed on the LA side throughout the day for the tap and first assist days (the participant did not attempt the independent exercise on any day). High levels of activity were observed on the LA side across all days of the study. The MA side showed much less activity overall with markedly lower acceleration magnitudes than on the LA side. During the cued exercise windows on Day 1 (tap), the participant generated higher magnitude accelerations with the LA arm, which was used to tap (and generate low magnitude accelerations from) the activity monitor on the MA side. During the cued exercise windows on Day 2 (ast), the participant generated acceleration profiles that exhibited more balance in the magnitude of accelerations recorded from the two arms.

A summary of activity counts across all 5 study days (Fig. 7B) shows that this participant rarely used the LA arm alone, whether during cued exercise windows (tap, assisted) or during non-cued, silent monitoring periods. The participant did move the MA and LA arms together 22% of the time during the cued assisted exercise windows on Days 2 to 5, which substantially exceeded the relative amount of time spent performing coincident motions during the cued exercise windows on Day 1 (i.e., 6%) and during the silent monitoring periods (< 1%). These observations were further supported by the MA:LA arm use ratios; the participant did not record any period with a use ratio greater than or equal to one, which reflects a strong bias towards use of the LA arm. Nevertheless, the participant did increase their use of the MA arm relative to the LA arm during the cued exercise windows as the challenge level of the exercise increased (i.e., from tapping to assisted elbow flexion/extension; Fig. 7C, left). Here again, a modest increase in MA arm use during non-cued periods on days when the participant performed the assisted exercise suggests a possible carry-over effect of the cued exercises on arm use into unstructured everyday activities.

The bilateral activity density plots of Fig. 7D further confirmed the participant’s bias towards use of the LA arm throughout the study. When wrist acceleration data were aggregated across all non-cued silent monitoring periods (non), the densest and most frequent instances of bilateral activity occurred at very low bilateral magnitudes and mainly to the left of the origin on the x-axis. During the tapping stage, stronger accelerations were recorded on the LA side with most of the instances of high bilateral magnitude occurring far to the left of the origin on the negative x-axis. Instances of positive bilateral use ratios (i.e., mostly MA side movement) occurred only at very low levels of bilateral magnitude. Even though the “hot spot” of bilateral arm activity shifted to the right during the cued assisted exercise windows (thereby reflecting greater balance between MA and LA arm use during those times) the overall pattern of arm activities remained biased toward negative use ratio values. This participant never achieved symmetry about the vertical line corresponding to a magnitude ratio of 0. Perhaps greater symmetry in arm use could have been achieved for some monitoring period if the participant had been able to perform the independent elbow flexion/extension exercise.

## Discussion and conclusions

This study examined the objective utility and subjective user experience of a personal exercise cueing system and progressive-challenge cued exercise program designed to monitor and motivate upper extremity activity in unstructured home settings. A small cohort of participants in the chronic stage of recovery from stroke engaged in a 5-day program of progressive-challenge exercise that was tailored to their individual capabilities and personal schedules. Most participants were able to follow the default three-stage program, which gradually increased the level of exercise difficulty over the first 3 days. Each participant also successfully self-scheduled each day’s exercise periods using a mobile phone application specifically designed for ease of use. The system had utility in the sense that it successfully delivered haptic exercise cues per the participants’ self-scheduled exercise windows and that the system effectively instigated upper extremity movements in response to each cue. Arm activity in the 5-s window immediately following each cue was greater than activity just before the cue, independent of the baseline activity level, which varied considerably across participants. The system also reliably monitored and recorded upper-limb activity over the course of each 8-h day. The system elicited positive user experiences in the sense that responses on surveys of usability, intrinsic motivation, and user satisfaction were generally positive; participants felt motivated by the system and found value in performing the cued exercises, they found the wearable system easy to use, and they were satisfied with the wearable devices and mobile interface. Although an assessment of system efficacy was beyond the scope of the current feasibility study, the recorded accelerometry data presented as case studies suggest potential benefits that might accrue from a program of progressive-challenge exercise in the chronic stage of stroke recovery for some stroke survivors. As was shown for the two selected participants, accelerometry data accurately reflected the graded challenge of the instructed exercises across the three progression stages: the “Independent” exercise elicited greater affected-side arm use than the “Assisted” exercise, which in turn elicited greater affected-side arm use than the “Tapping” exercise. Intriguingly, the pattern of increasing arm use on days with increased exercise challenge appeared to spill-over into unstructured activities performed throughout the rest of the participants’ days (i.e., outside the cued exercise windows). Future studies will be required to assess the exercise program’s potential to enhance functional recovery through activity cueing and monitoring during all stages of recovery.

### Accelerometry as a means to assess adherence and its impact on daily activity

Home exercise programs are a critical component of post-stroke care because benefits from exercise continue to accrue even beyond the acute stage of recovery and dedicated periods of in-clinic therapy [56, 57]. The maximum benefits of these programs are not often fully understood because they typically are performed outside of clinical supervision. In some cases, potential benefits remain unrealized due to patients not exercising as prescribed. A systematic review of clinical trials involving mobile health applications designed with the intent to improve physical activity outcomes in stroke rehabilitation indicated that few studies (36%) reported user adherence data, while those that did often showed mixed- or low-level adherence [58]. Broadly speaking, patient adherence to unsupervised home exercise programs following discharge from clinical care is low; some surveyed populations have reported non-adherence rates up to 35%, with patients performing different exercises than those prescribed being cited as a top reason for non-adherence [6]. There is a strong need for valid and direct measurements of adherence to specific exercise programs because self-reported methods are not reliable and have been shown to overestimate or underestimate the actual amount of exercise performed [59]. Accelerometry data from wearable sensors have potential to give clinicians valuable insight into patient exercise behavior by providing accurate real-time activity assessments that are resistant to biases observed in self-report methods [59]. Indeed, using accelerometry to quantify upper extremity activity in the stroke patient population has been well validated as a viable approach [22, 60, 61].

In the current study, we obtained further support for the idea that upper-extremity accelerometry data can quantify adherence to specific at-home exercise protocols. By comparing cue response rates relative to background activity rates (within 5-s of the cues), we found strong adherence to the prescribed exercises (98% ± 3.1%). Further, by providing participants with a set of graded-challenge exercises to perform across days, the two case studies presented here demonstrate how the collection of synchronized measures of unilateral arm activity enable inference regarding adherence to specific bimanual arm exercises. The difference in arm activity levels across exercise types was evident when we considered each arm’s accelerometry data together and in isolation (Figs. 6B and 7B). The percentages of single-arm and combined-arm usages reflect exactly the expected result for each exercise, with tapping exercise showing predominant use of the LA arm, the assisted exercise showing mostly bilateral arm use, and the independent exercise eliciting predominant use of the MA arm. We found the ratio of isolated arm usage between the affected and unaffected arms (Figs. 6C and 7C) to be especially valuable because this measure discounts activity or motion common to both sides, such as may occur during walking. Dramatic differences in the cued use ratio between the tapping and independent exercise periods reflect expected asymmetrical usages for each exercise type. We acknowledge however that future application of at-home exercise monitoring technology will require customization of activity monitoring algorithms to the specific exercises prescribed to address each patients’ particular needs.

A specific strength and novelty of our study stems from the quantifiable insight gained from combining a graded exercise program with accelerometer-based monitoring that extends beyond scheduled exercise periods. This is demonstrated most clearly in the case studies presented in Figs. 6 and 7, where large differences in cued arm use were noted between the easiest exercise (tapping) and the more challenging exercises (assisted and independent). The fact that similar trends in arm use were also observed during non-cued silent monitoring periods in both the less-impaired participant (Fig. 6) and the most impaired participant (Fig. 7) suggests a potential benefit of graded-challenge exercise on arm use throughout the day regardless of baseline impairment levels.

### Impact of subjective user experience

For at-home exercises to be effective, patients must in fact perform them. The wearable cueing system and progressive-challenge exercise program described here was designed to leverage the computing power of a smartphone and low-cost wearable activity monitors to overcome forgetfulness, which is a common obstacle to performance of home exercise programs. Adherence to prescribed home-based therapies is strongly linked to users having a positive experience with the intervention [62, 63]. Positive interactions between a user and a mobile health system derives from a broad range of factors including (but not limited to) ease of use within the intended use scenario, ease of learning, ease of troubleshooting when issues arise, ability to elicit personal motivation, and overall satisfaction with the technology. Quantifying user experience with supporting technology in the context of its specific use scenario is needed therefore to understand its potential impact on both adherence and efficacy of an at-home exercise program.

Average responses on the System Usability Scale were high (84 ± 8.0 out of 100) indicating “excellent” overall system usability [64] of our exercise cueing system in the context of a progressive-challenge exercise program. This was a critical benchmark to reach because even applications with high utility may not find user acceptance if usability is poor [63]. Participants also reported significantly positive user satisfaction scores on the QUEST survey, both for the devices and for the training materials that described the system’s setup, use, and troubleshooting. Patient satisfaction is an important component of user experience because it can predict usage of health services [65]. According to participants, the top three most important factors contributing to satisfaction with our system and exercise program were *comfort*, *effectiveness*, and *provided instructions*. The fact that we used a user-centered design process with iterative modifications to ensure participants would feel comfortable wearing the devices and engage in the cued exercise program likely contributed to the high satisfaction scores. Further, our cued exercise program was clinician-designed to engage patients across a broad range of impairment levels. The easiest activity required absolutely no ability to move the more involved arm, which likely contributed to the high levels of perceived self-efficacy we report. We also attended carefully to providing an easy-to-follow graphical instruction manual because stroke is more common in older adults and older individuals experience more anxiety related to technology use than do younger people [66]. Satisfaction with instructions in home-based therapy is crucial because users must feel confident managing the technology independently given the limited support clinicians can offer due to current healthcare reimbursement constraints. Understanding the efficacy of support offered to participants both indirectly through the training material and directly through phone calls will be of particular importance as it has been shown that frequent check-ins from therapists in other home-based exercise programs can effectively improve outcomes [67].

Finally, the intensity of therapeutic exercise can be higher when motivation is also high, stressing the importance for patients to find intrinsic motivation when performing home-based exercise therapy [68] (see also [12]). Participants in our study reported positive outcomes on five of the IMI subcategories: *perceived competence, value, choice, pressure, and effort*. Overall, *perceived competence* and *value* ranked as the top two subcategories, indicating both that participants felt confident in their ability to use the wearable system at home without daily intervention from a clinician and that they also recognized the personal value in adding the exercises to their daily routines. These findings demonstrate that our participants derived intrinsic motivation from using the system in the context of a progressive-challenge cued exercise program at-home in the absence of concurrent clinician guidance. Although the average *interest* subcategory score did exceed the positive threshold value, some participants evidently found the cued exercises more engaging than others. This may have reflected the fact that the cued exercises were rather easy for some participants (but not all). Future studies may want to find a way to increase user interest so as to improve the overall user experience and potential benefit of the cued exercise program as a whole.

### Limitations and future directions

The purpose of our study was not to compare our wearable system to other rehabilitation devices, but rather to assess the utility and usability of our wearable system within the specific context of an at-home program of graded-challenge cued exercise. Because the study did not focus on efficacy of the graded-challenge cued exercise program, the lack of control participants was not a limitation. Likewise, it was not a limitation that our participants could take different trajectories through the sequences of exercises across days (Table 3). This possibility was allowed by the experimental design and demonstrates how the system can elicit cued exercises with a difficulty level graded to each individual’s motor capacity across a diverse pool of participants.

Although the study’s sample size was small and spanned a wide range of participant ages (29 to 63 years) and number of years post-stroke (2 to 21 years), we found clear support for utility in the sense that the wearable system successfully delivered haptic exercise cues per the participants’ self-scheduled exercise windows. However, given that we encountered a 25% drop-out rate from the study, the use of a wearable exercise cueing system at-home may not be suited for every patient; further studies should identify factors that contribute favorably (or unfavorably) to the engagement in cued exercise programs such as the one described in this report.

For the individuals who did complete the entire study protocol, the system provided strongly positive user experiences (Fig. 5). We acknowledge that the support offered by the researchers (i.e., up to two daily check-in phone calls) may have motivated participants to adhere to the study protocol and that such frequent communication is probably impractical to replicate in an outpatient rehabilitation setting. Although it is difficult to control for participant motivation, it is unlikely that the results we report derived mainly from a general increase in arm activity due to the check-in phone calls; the results in Fig. 4 demonstrate cue specificity in the form of a marked increase in arm activity in the 5-s periods immediately following cues relative to the 5-s periods immediately preceding the cues. It is also worth noting that the majority of participants did not continue the check-in calls beyond the first day or two. A related limitation of our study was the lack of direct real-time feedback of exercise performance provided to participants. It is recognized that goal setting and regular feedback can be effective in motivating participants to engage in rehabilitation programs [69]. Participants in future studies may benefit from receiving direct in-the-moment feedback regarding exercise performance from within the smart-phone application, which may in turn increase motivation and engagement.

Another limitation of this study was its focus on an at-home setting with all participants in the chronic stage of recovery; other previous work also has shown significant benefits to arm function from self-directed interventions more than 12 months poststroke [70]. We suggest that additional studies with the wearable system should be performed during acute and subacute recovery to better understand the potential benefits of self-directed cued exercise, and to monitor its impact on arm usage throughout the early phases of stroke recovery. A related limitation was our use of a single dosage of cued exercise (1.5 h of cued exercise per day over the 5 day protocol) and just one specific set of exercises. It is possible that greater improvement in hemiparetic arm use might accrue with a different dosage or combination of different cued exercise [71, 72]. Prospective dose-finding studies are needed to assess the impact of specific cued exercises on hemiparetic arm use and on the overall user experience throughout all phases of recovery.

### Supplementary Information

**Additional file 1: Table S1.** Survey results by participant.

## Data Availability

The datasets used and/or analyzed during the current study are available from the corresponding author on reasonable request.

## Abbreviations

tap: The TAPPING exercise
ast: The ASSIST exercise
ind: The INDEPENDENT exercise
LA: Less-affected ipsilesional arm
MA: More-affected contralesional arm
SUS: System Usability Scale
IMI: Intrinsic Motivation Inventory
QUEST: Quebec User Evaluation of Satisfaction with assistive Technology
SD: Standard deviation
SEM: Standard error of the mean
