# Supplementary material for: Utility and usability of a wearable system and progressive-challenge cued exercise program for encouraging use of the more involved arm at-home after stroke—a feasibility study with case reports
Source: J Neuroeng Rehabil. 2024 Apr 30;21:66. doi: 10.1186/s12984-024-01359-0 (PMC11059679; doi:10.1186/s12984-024-01359-0)
Supplement: Supplementary file 1 — Additional file 1: Table S1. Survey results by participant. [file 12984_2024_1359_MOESM1_ESM.docx]

**Table S1:** Survey results by participant

|  | **SUS** | **QUEST** | | **IMI** | | | | | |
| --- | --- | --- | --- | --- | --- | --- | --- | --- | --- |
| **Participant** | **Composite** | **Devices** | **Services** | **Competence** | **Pressure** | **Effort** | **Choice** | **Interest** | **Value** |
| 1 | 95 | 4.8 | 4.6 | 7.0 | 1.6 | 7.0 | 5.3 | 6.3 | 7.0 |
| 2 | 80 | 5.0 | 4.0 | 5.7 | 3.4 | 6.8 | 5.0 | 5.1 | 6.7 |
| 3 | 93 | 4.8 | 4.8 | 7.0 | 2.4 | 7.0 | 5.1 | 4.7 | 6.1 |
| 4 | 80 | 4.8 | 4.0 | 3.8 | 2.8 | 4.6 | 4.9 | 2.3 | 6.1 |
| 5 | 80 | 5.0 | 4.8 | 7.0 | 2.2 | 4.2 | 5.0 | 3.3 | 3.4 |
| 6 | 75 | 4.5 | 5.0 | 5.8 | 2.6 | 5 | 3.7 | 3.7 | 6.3 |
| **mean (SD)** | **83.8 (8.0)** | **4.8 (0.2)** | **4.5 (0.4)** | **6.1 (1.3)** | **2.5 (0.6)** | **5.8 (1.3)** | **4.8 (0.6)** | **4.2 (1.4)** | **6.0 (1.3)** |

**Abbreviations:** SUS: system usability scale; QUEST: Quebec user evaluation of satisfaction with assistive technology; IMI: intrinsic motivation inventory; SD: standard deviation
